# Next-generation sequencing methylation profiling of subjects with obesity identifies novel gene changes

**DOI:** 10.1186/s13148-016-0246-x

**Published:** 2016-07-18

**Authors:** Samantha E. Day, Richard L. Coletta, Joon Young Kim, Latoya E. Campbell, Tonya R. Benjamin, Lori R. Roust, Elena A. De Filippis, Valentin Dinu, Gabriel Q. Shaibi, Lawrence J. Mandarino, Dawn K. Coletta

**Affiliations:** School of Life Sciences, Arizona State University, Tempe, AZ USA; School for the Science of Health Care Delivery, Arizona State University, Phoenix, AZ USA; Division of Weight Management and Wellness Children’s Hospital of Pittsburgh, Pittsburgh, PA USA; Endocrinology Department, Mayo Clinic in Arizona, Scottsdale, AZ USA; The Department of Biomedical Informatics, Arizona State University, Phoenix, AZ USA; College of Nursing and Health Innovation Arizona State University, Phoenix, AZ USA; Mayo/ASU Center for Metabolic and Vascular Biology, Mayo Clinic in Arizona, Scottsdale, AZ USA; Division of Endocrinology, Diabetes and Metabolism in the Department of Medicine at the UA College of Medicine, University of Arizona, Tucson, AZ USA; School of Nutrition and Health Promotion, College of Health Solutions, Arizona State University, 550 N. 3rd Street, Phoenix, AZ 85004 USA; Department of Basic Medical Sciences, The University of Arizona College of Medicine, Phoenix, AZ USA

**Keywords:** Methylation, Next-generation sequencing, Skeletal muscle, Obesity

## Abstract

**Background:**

Obesity is a metabolic disease caused by environmental and genetic factors. However, the epigenetic mechanisms of obesity are incompletely understood. The aim of our study was to investigate the role of skeletal muscle DNA methylation in combination with transcriptomic changes in obesity.

**Results:**

Muscle biopsies were obtained basally from lean (*n* = 12; BMI = 23.4 ± 0.7 kg/m^2^) and obese (*n* = 10; BMI = 32.9 ± 0.7 kg/m^2^) participants in combination with euglycemic-hyperinsulinemic clamps to assess insulin sensitivity. We performed reduced representation bisulfite sequencing (RRBS) next-generation methylation and microarray analyses on DNA and RNA isolated from *vastus lateralis* muscle biopsies. There were 13,130 differentially methylated cytosines (DMC; uncorrected *P* < 0.05) that were altered in the promoter and untranslated (5' and 3'UTR) regions in the obese versus lean analysis. Microarray analysis revealed 99 probes that were significantly (corrected *P* < 0.05) altered. Of these, 12 genes (encompassing 22 methylation sites) demonstrated a negative relationship between gene expression and DNA methylation. Specifically, sorbin and SH3 domain containing 3 (SORBS3) which codes for the adapter protein vinexin was significantly decreased in gene expression (fold change −1.9) and had nine DMCs that were significantly increased in methylation in obesity (methylation differences ranged from 5.0 to 24.4 %). Moreover, differentially methylated region (DMR) analysis identified a region in the 5'UTR (Chr.8:22,423,530–22,423,569) of SORBS3 that was increased in methylation by 11.2 % in the obese group. The negative relationship observed between DNA methylation and gene expression for SORBS3 was validated by a site-specific sequencing approach, pyrosequencing, and qRT-PCR. Additionally, we performed transcription factor binding analysis and identified a number of transcription factors whose binding to the differentially methylated sites or region may contribute to obesity.

**Conclusions:**

These results demonstrate that obesity alters the epigenome through DNA methylation and highlights novel transcriptomic changes in SORBS3 in skeletal muscle.

**Electronic supplementary material:**

The online version of this article (doi:10.1186/s13148-016-0246-x) contains supplementary material, which is available to authorized users.

## Background

Obesity is a condition that affects about one third of the US adult population [1]. It is a major disease associated with other co-morbidities, including type 2 diabetes, metabolic syndrome, and cardiovascular disease [2]. An underlying feature of obesity is insulin resistance. Insulin resistance is a reduced biological response of insulin on peripheral tissues including skeletal muscle, liver, and fat [3]. Under normal physiological conditions, skeletal muscle accounts for approximately 80 % of insulin-stimulated total body glucose uptake [4]. Previous studies from our laboratory have investigated the molecular mechanisms of insulin resistance in skeletal muscle. We have previously shown that insulin resistance in skeletal muscle is in part due to mitochondrial dysfunction [5]. In experimentally induced insulin resistance, we have shown a low grade inflammatory response, with increases in extracellular matrix (ECM) turnover [6]. Furthermore, by using a proteomic approach on insulin resistant muscle, we identified alterations in the abundance of protein involved in cytoskeletal structure and assembly [7]. Our findings, to date, demonstrate a cross talk relationship between inflammation, extracellular remodeling, cytoskeletal interactions, mitochondrial function, and insulin resistance in human skeletal muscle [8].

The pathogenesis of obesity-associated insulin resistance is due to environmental and genetic factors [9, 10]. However, the role of epigenetic factors, which may provide a potential link between the genetic and environmental factors observed in obesity, is poorly understood. Epigenetics can be described as heritable changes in gene function that occur without a change in nucleotide sequence [11]. DNA methylation is an epigenetic modification and is generally observed as a methyl addition to the carbon 5 position of cytosines and more commonly on cytosines preceding guanines, called CpG dinucleotides [12]. DNA methylation patterns are established during early development and are maintained in differentiated tissue by DNA methyltransferases [13]. Changes in DNA methylation are a potential mechanism by which the expression of a gene may be regulated [12]. For example, it is generally accepted that gene expression is often reduced when DNA methylation is present at a promoter or untranslated region of a gene [14–16].

There have been a number of studies that have focused on the epigenetic basis of obesity [17, 18]. However, the majority of the DNA methylation studies performed to date have either used a candidate gene approach or the array based technology that probes 450K methylation sites simultaneously. Therefore, our study is unique in that we performed reduced representation bisulfite sequencing (RBBS), which has the ability to capture millions of methylation sites in the human genome. Moreover, we performed transcriptomic analyses, which allowed us to measure global messenger RNA (mRNA) expression levels in genes altered in people with obesity. Furthermore, we combined epigenetic and transcriptomic analyses to identify associations between the datasets. Based on our previous findings in skeletal muscle, we hypothesize that there will be alterations in the methylation of genes involved in mitochondrial function, inflammation, and extracellular matrix remodeling.

## Methods

### Participants

Ten insulin resistant participants with obesity and 12 insulin sensitive participants without obesity were recruited. Insulin sensitivity was assessed by the euglycemic-hyperinsulinemic clamp [19]. Demographic, medical history, anthropometric, metabolic, and screening blood tests were obtained on all participants. Percent body fat was assessed by body impedance analysis. Normal glucose tolerance was assessed by a 75-g oral glucose tolerance test following a 10–12 h overnight fast. No subject was taking any medication known to affect glucose metabolism. All subjects gave informed written consent to participate in the study, which was approved by the Institutional Review Boards of the Mayo Clinic in Arizona and Arizona State University.

### Study design

Following an overnight fast, participants reported to the Clinical Studies Infusion Unit at the Mayo Clinic in Arizona. A 2 h euglycemic-hyperinsulinemic clamp (80 mU m^−2^ min^−1^) was performed [19]. A primed infusion of 6,6 di-deuterated glucose was begun at −120 min to determine the basal rate of glucose metabolism. Sixty minutes after the start of deuterated glucose infusion, a resting, basal *vastus lateralis* muscle biopsy was performed percutaneously, under local anesthesia, as previously described [19, 20]. After resting for 1 h, a primed continuous infusion of insulin was started. The constant infusion of deuterated glucose was discontinued at time 15 min after the start of the insulin infusion, and a variable infusion of 20 % dextrose that was enriched with 6,6 di-deuterated glucose was used to maintain euglycemia and a constant enrichment of the tracer. Enrichment of plasma glucose with 6,6 di-deuterated glucose was assayed using GC/MS in the Center for Clinical and Translational Science (CCaTS) Metabolomics Core at the Mayo Clinic in Rochester. The rates of glucose appearance and disappearance were calculated using steady state equations to derive insulin sensitivity levels, termed the *M* value [21].

### Substrate and hormone determinations

Plasma glucose concentration was determined by the glucose oxidase method on an YSI 2300 STAT plus (YSI INC., Yellow Springs, OH, USA). Plasma insulin was measured by a two-site immunoenzymatic assay performed on the DxI 800 automated immunoassay system (Beckman Instruments, Chaska, MN, USA). Inter-assay C.V.s were 6.2 % at 5.3 uU/mL, 6.5 % at 46.1 uU/mL, and 7.7 % at120.4 uU/mL. A comprehensive metabolic panel, lipid panel, and hemogram panel were performed by the Biospecimens Accessioning and Processing (BAP) Core at the Mayo Clinic in Scottsdale.

### Muscle biopsy processing

For genomic DNA analyses, homogenization of the muscle biopsy (25 mg) was performed in 1× PBS with the Bullet Blender (Integrated Scientific Solutions, San Diego, CA). DNA was isolated using QIAamp DNA mini kit, as per the manufacturer’s instructions (Qiagen, Valencia, CA). For mRNA analyses, muscle biopsy specimens (50 mg) were homogenized in TRIzol solution (Invitrogen, Carlsbad, CA) using a Polytron (Brinkmann Instruments Westbury, NY). Total RNA was purified with RNeasy MinElute Cleanup Kit (Qiagen, Chatsworth, CA). DNA and RNA quality and quantity were determined using gel electrophoresis and A260/A280 values.

### Reduced representation bisulfite sequencing (RRBS)

RRBS was performed at the Mayo Clinic Genotyping Shared Resource facility as previously described [22]. DNA (250 ng) was digested with Msp1 (New England Biolabs, Ipswich, MA) and purified using QIAquick Nucleotide Removal Kit (Qiagen, Valencia, CA). End-repair A tailing was performed (New England Biolabs, Ipswich, MA) and TruSeq methylated indexed adaptors (Illumina, San Diego, CA) were ligated with T4 DNA ligase (New England Biolabs, Ipswich, MA). Size selection was performed with Agencourt AMPure XP beads (Beckman Coulter, Indianapolis, IN). Bisulfite conversion was performed using EZ DNA Methylation Kit (Zymo Research, Irvine, CA) as recommended by the manufacturer with the exception that an incubation was performed using 55 cycles of 95 °C for 30 s and 50 °C for 15 min. Following bisulfite treatment, the DNA was purified as directed and amplified using Pfu Turbo C Hotstart DNA Polymerase (Agilent Technologies, Santa Clara, CA). Library quantification was performed using Qubits dsDNA HS Assay Kit (Life Technologies, Grand Island, NY) and the Bioanalyzer DNA 1000 Kit (Agilent Technologies Santa Clara, CA). The final libraries from RRBS were placed onto seven lanes of a paired-end flow cell at concentrations of 7–8 pM, and the control sample, PhiX, was placed in the eighth lane to allow the sequencer to account for the unbalanced representation of cytosine bases. The flow cell was then loaded into the Illumina cBot for generation of cluster densities. After cluster generation, the flow cells were sequenced as 51 × 2 paired end reads using Illumina HiSeq 2000 with TruSeq SBS sequencing kit version 3. Data was collected using HiSeq data collection version 1.5.15.1 software, and the bases were called using Illumina’s RTA version 1.13.48.

### RRBS data analysis

RRBS data was analyzed using a streamlined analysis and annotation pipeline for reduced representation bisulfite sequencing, SAAP-RRBS [23]. FASTQ were trimmed to remove adaptor sequences, and any reads with less than 15 base pair (bp) were discarded. Trimmed Fastqs were then aligned against the reference genome Hg19 using BSMAP [24], which converts the reference genome to align the bisulfite-treated reads. Samtools was used to get mpileup, and PERL scripts as described elsewhere [23] were used to determine CpG methylation and non-CpG methylation to estimate the bisulfite conversion efficiency [25]. Methylation ratios were reported along with custom CpG annotation. The methylation dataset supporting the conclusions of this article are available in the Gene Expression Omnibus repository, GSE73304 (http://www.ncbi.nlm.nih.gov/geo/). Additionally, bigwig files were used to create a custom track on the UCSC genome browser (https://genoe.ucsc.edu/cgi-bin/hgTracks?hgS_doOtherUser=submit&hgS_otherUserName=rlcolett&hgS_otherUserSessionName=testnoinitial).

### Differentially methylated cytosines (DMC) analysis

To determine differences in methylation between groups, the aligned data was imported into the free open source R package, methylSig. A minimum of five reads and the recovery of the site in at least eight participants from each group were required for the inclusion of a cytosine in downstream analyses. The mean methylation differences (%) between the groups with and without obesity were adjusted by a beta binomial approach to account for biological variation among the groups being compared [26]. A comparison of the DNA methylation between groupings at each site was based on a likelihood ratio test (nominal Pvalue), and a Benjamini-Hochberg multiple testing correction was applied. Benjamini-Hochberg correction yielded no significant sites; therefore, for subsequent analyses, an uncorrected *P* < 0.05 was used. The RefSeq Genes and CpG Island tracks from the University of California, Santa Cruz (UCSC) Genome Browser were imported for additional region annotations. When applying regional annotation to each DMC, priority was given to annotating the site as a promoter or untranslated region if that site was in another transcript of the gene or in a different gene.

### Differentially methylated region (DMR) analysis

DMRs were identified using the open source R package dispersion shrinkage for sequencing data (DSS) [27]. The BSmooth algorithm was applied to the entire data set to determine the level of methylation in a region for each sample and to account for biological variation. The following criteria were used for the analysis: each region contained two CpGs supported with a read coverage of 5×, the recovery of the site in at least eight participants from each group, and significance of *P* < 0.05 from the DMC analysis. DMRs were created based on a t-statistic cutoff of 2.5 and a sliding-window of 500 bp. The significance of a DMR was weighted by the Area Stat, which is the sum of t-statistic values in each DMR. Additional region annotations were included by importing RefSeq Genes and CpG Island tracks from the UCSC Genome Browser into the R package, Genomic Ranges. When applying regional annotation to each region, priority was given to annotating the region as a promoter or untranslated region if the sites were in another transcript of the gene or in a different gene.

### Microarray processing

Total RNA (100 ng) was amplified and labeled using the Low Input Quick Amp Labeling Kit, One-Color, as per manufacturer’s instructions (Agilent Technologies, Santa Clara, CA). After labeling, complimentary RNA (cRNA) was fragmented using Agilent Gene Expression Hybridization Kit (Agilent Technologies, Santa Clara, CA), as per instructions. The fragmented cRNA was hybridized to the SurePrint G3 Human Gene Expression 8x60K v2 Microarray (Agilent Technologies, Santa Clara, CA) using a SureHyb DNA Microarray Hybridization Chamber at 65 °C, for 17 h in a rotating incubator. After hybridization, slides were washed in Gene Expression wash buffers 1, 2, and acetonitrile as per instructions, and then scanned with an Agilent DNA microarray scanner (Agilent Technologies, Santa Clara, CA).

### Microarray analysis

Feature Extraction Software version 12.0.1.1 (Agilent Technologies, Santa Clara, CA) was used for the array image analysis. The microarray dataset supporting the conclusions of this article are available in the Gene Expression Omnibus repository, GSE73078 (http://www.ncbi.nlm.nih.gov/geo/). The data files were imported into the free open source R package, Linear Models for Microarray Data (Limma) version 3.22.0 (http://www.bioconductor.org/packages/release/bioc/html/limma.html). Data were background corrected using normal exponent, quantile normalized, and an unweighted linear model was performed to generate fold changes between groups. The fold changes were log transformed. Expression values obtained were evaluated by a moderated t-statistic (nominal *P* value) and adjusted using the Benjamini-Hochberg multiple testing correction.

### SORBS3 DMC site specific validation

DNA methylation was assessed using a site specific sodium bisulfite sequencing method. DNA (500 ng) was treated with sodium bisulfite using the EZ DNA Methylation-Lightening kit (Zymo Research, Irvine, CA). Chromosome 8 (Chr.8) positions 22,422,428–22,422,868 proximal to the transcription start site for SORBS3 was amplified by PCR using the following primers: forward 5′-AGAGATATAATTTGGTAG AAATTGGTAGGATTG-3′, reverse 5′AATTACCCGCAAATCCTTATCCAAC-3′ (342 bp). The cycling conditions were 95 °C for 10 min followed by 40 cycles of 95 °C for 30 s, 56 °C for 40 s, and 72 °C for 1 min with touchdown annealing temperatures for the first 10 cycles, and a final extension at 72 °C for 7 min. The products were run on a 1 % agarose gel with ethidium bromide and ultraviolet detection. The 342 bp product bands were purified using Zymoclean Gel DNA Recovery Kit, per the manufacturer’s instructions (Zymo Research, Irvine, CA). Sanger Sequencing was performed on the bisulfite-converted forward DNA strands at Arizona State University’s Sequencing Core. The proportion of methylation on each CpG site was detected using the Epigenetic Sequencing Methylation analysis software (ESME).

### SORBS3 DMR pyrosequencing validation

To confirm DNA methylation of the chromosome 8 region 22,423,530–22,423,569, pyrosequencing PCR and sequencing primers were designed using the PyroMark Assay design Software 2.0 (Qiagen, Valencia, CA). The forward and reverse primers were biotinylated at the 5′ end. Bisulfite conversion of 500-ng genomic DNA was performed using the EZ DNA Methylation-Lightening kit according to the manufacturer’s instructions (Zymo Research, Irvine, CA). To assess the forward strand, bisulfite-converted DNA was amplified by PCR using the following primers: forward 5′-AGTAGGGGGAGGAAGGAA-3′ and biotinylated reverse 5′- ACTCTCCACAAAATATCCTACTTC-3′. To assess the reverse strand, bisulfite-converted DNA was amplified by PCR using the following primers: biotinylated forward 5′-AGTAGGGGGAGGAAGGAA-3 and reverse 5′-ACCCCCATCCTCTACTAAAAATTAACTACC-3′. Pyrosequencing was performed using the PyroMark Q96 MD system and the Gold Q96 kit with sequencing primers: 5′-GTGTTAGGGAGGGAT-3′ (forward strand assessment) and 5′-CTACTAAAAATTAACTACCCTC-3′ (reverse strand assessment) according to the manufacturer’s instructions (Qiagen, Valencia, CA). Data analysis was performed using the PyroMark CpG SW 1.0 software (Qiagen, Valencia, CA).

### SORBS3 qRT-PCR validation

Skeletal muscle gene expression for SORBS3 was detected using quantitative real-time PCR on the ABI PRISM 7900HT sequence detection system (Life Technologies, Carlsbad, CA). TaqMan Universal Fast PCR master mix reagents and the Assay-On-Demand gene expression primer pair and probes (Life Technologies, Carlsbad, CA) were added to 20 ng cDNA, which was synthesized using the ABI High Capacity cDNA Reverse Transcription Kit, as per manufacturer’s instructions. The quantity of SORBS3 (Hs00195059_m1) in each sample was normalized to 18S (Hs99999901_s1) using the comparative (2-∆∆CT) method [28].

### SORBS3 predicted transcription factor binding analysis

Transcription factor binding sites analysis was performed using PROMO version 3.0.2 [29]. The sequences were analyzed with a 5 % maximum matrix dissimilarity rate using TRANSFAC version 8.3 database. Analysis of the nine SORBS3 DMCs was assessed as three separate sequences: Chr.8: 22,409,277–22,409,317; Chr.8: 22,422,628–22,423,112; and Chr.8: 22,423,280–22,423,363. Furthermore, the SORBS3 DMR sequence Chr.8:22,423,530–22,423,569 was assessed for transcription factor binding sites.

### Statistical analysis

Participant characteristic data was presented as a mean ± SEM, and comparisons between the groups with and without obesity were based on an independent sample *t* test. Non-normally distributed data for the 2 h insulin were log10 transformed; however, untransformed data are presented for ease of interpretation. Analysis of covariance (ANCOVA) was used to adjust for the effects of age, sex, and the interaction between age and sex. PASW version 22.0 was used for the characteristic data analyses with the significance set at *P* ≤ 0.05. Pearson correlation was used for all correlations presented. See above for the statistical analysis of the methylation and microarray data.

## Results

### Participants

Table 1 shows the phenotypic characteristics for participants with and without obesity. There was a significant age difference between groups whereby, individuals with obesity were older. By design, the lean participants had significantly lower body mass index (BMI), body fat, and waist circumference. The participants with obesity were significantly more insulin resistant compared to the lean group, determined by the *M* value. These differences remained significant after adjusting for potential covariates including age, sex, and the interaction between age and sex.

**Table 1:** Characteristics of study participants (*n* = 22) classified by body mass index

Characteristics	Lean	Obese	*P* value	*P* value (age, sex, age × sex)
Sex	7F/5M	4F/6M	NS^a^	–
Age (years)	28.8 ± 2.0	40.3 ± 2.5	<0.01	–
Body mass index (kg/m^2^)	23.4 ± 0.7	32.9 ± 0.7	<0.001	<0.001
Body fat (%)^b^	25.2 ± 1.4	35.2 ± 2.2	<0.001	<0.001
Waist circumference (cm)	82.0 ± 3.0	104.4 ± 2.5	<0.001	<0.01
Systolic blood pressure (mmHg)	119.8 ± 2.4	123.9 ± 3.1	NS	NS
Diastolic blood pressure (mmHg)	72.6 ± 1.5	78.2 ± 1.3	<0.05	NS
Triglycerides (mg/dL)	96.5 ± 13.3	114.7 ± 15.2	NS	NS
Cholesterol (mg/dL)	176.2 ± 9.2	186.1 ± 11.4	NS	NS
High-density lipoproteins (mg/dL)	57.1 ± 5.2	50.2 ± 3.4	NS	NS
Low-density lipoproteins (mg/dL)	99.9 ± 7.3	113.0 ± 10.3	NS	NS
Hemoglobin A1c (%)	5.2 ± 0.04	5.4 ± 0.1	NS	NS
Fasting plasma glucose (mg/dL)	86.7 ± 1.8	89.5 ± 1.7	NS	NS
2 h plasma glucose (mg/dL)	101.9 ± 5.2	111.2 ± 7.0	NS	NS
Fasting plasma insulin (μU/mL)	6.3 ± 1.1	11.1 ± 0.9	<0.01	NS
2 h plasma insulin (μU/mL)	43.2 ± 5.3	93.3 ± 16.4	<0.01	≤0.05
*M* value (mg/kg• min)	7.3 ± 0.6	4.5 ± 0.7	<0.01	<0.01

Data presented as mean ± SEM, based on independent sample *t* tests and two-tailed *P* values. Adjustment for age, sex, and the interaction of age × sex using ANCOVA

^a^Calculated by chi-square test

^b^Body fat determined by biometric impedance analysis (BIA)

### Global methylation analysis in human skeletal muscle

**Fig. 1:**
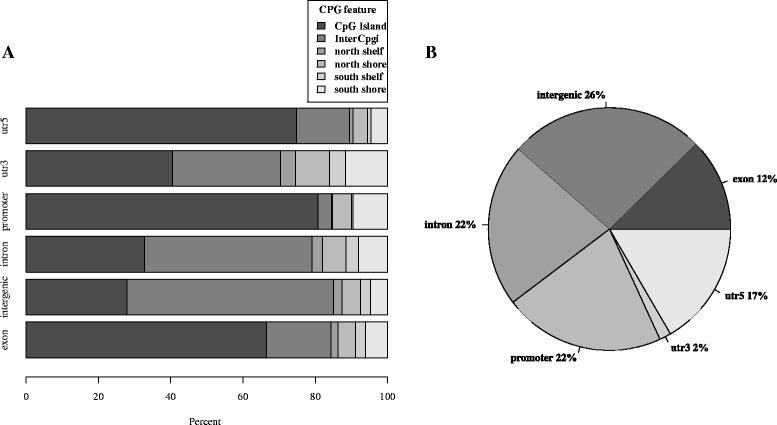
The methylation sites captured in our skeletal muscle samples using reduced representation bisulfite sequencing technology were mapped in the context of both gene regions (**a**) and CpG island features (**b**). The regions were defined using UCSC browser refGene and CpG island tracks (see the “Methods” section). The promoter region was defined as 1000 bp (basepairs) upstream of the transcription start site (TSS); untranslated region (UTR); CpG island is 200–3000 bp stretch of DNA with a C + G content of 50 % and observed CpG/expected CpG in excess of 0.6; North (N) and South (S) shores flank the CpG island by 0–2000 bp; the North (N) and South (S) shelf flank the shores by 2000 bp (2000–4000 bp from the island)

Prior to the quality control of the sequence data, 5,421,504 sites were captured using the RRBS technology. For our RRBS analysis, we set a threshold of greater than 80 % call rate and a minimum of 5× coverage for the sequencing data. Of the 22 participants sequencing data, 20 (11 lean and 9 obese) met this threshold criteria and were used for subsequent downstream analyses. For the sequencing data, we only included methylation sites that were captured in at least eight participants in each group. In total, we captured 2,586,085 methylation sites using these criteria. The distribution of the methylation sites was defined by genic regions (Fig. 1a) and CpG island features (Fig. 1b). We demonstrated that the majority of the methylation sites were in intronic regions (Fig. 1a). However, the sites in the promoter and 5′ untranslated regions (UTR) dominantly overlapped with CpG islands (Fig. 1b).

### Differentially methylated cytosine (DMC) analysis in promoter, 5′UTR, and 3′UTR regions

To investigate the sites that may generate the greatest changes in mRNA expression based on proximity, we sought sites in untranslated regions (5′ and 3′UTR) and assigned our promoter region as 1000 base pairs from the transcription start site region (0 to −1000 base pairs). Of the 2,586,085 methylation sites captured, 710,981 sites were located in our defined proximal regions and 13,130 of those sites were significantly altered (nominal *P* < 0.05; Additional file 1: Table S1) between our groupings. Differentially methylated cytosines (DMCs) between the groupings were assessed for false discoveries. There were no sites that met the criteria of a false discovery rate *P* < 0.05. As such, we used nominal *P* value cutoffs, which have been accepted in other studies [14, 30].

### Overlying changes between DNA methylation and gene expression

**Fig. 2:**
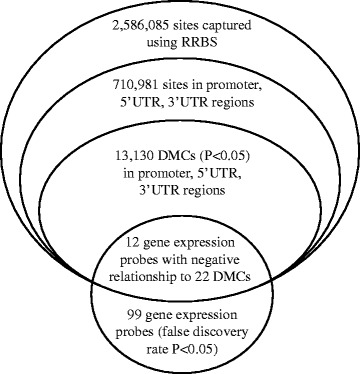
Diagram of the analysis for differentially methylated cytosines (DMCs) localized in a promoter, 5' UTR, or 3'UTR region overlapping with transcriptomic changes

Transcriptomic analysis identified 99 probes that were significantly (false discovery rate *P* < 0.05) altered in the group with obesity (Additional file 2: Table S2). We compared the significant genes identified from our microarray analysis with the significant DMCs that were found in the promoter, 5′UTR, and 3′UTR (*n* = 13,130; *P* < 0.05; Fig. 2). We identified 12 genes (encompassing 22 methylation sites) that demonstrated a negative relationship between gene expression and DNA methylation. Of these, sorbin and SH3 domain containing 3 (SORBS3) had increased methylation (9 DMCs) and was associated with a decrease in gene expression. The 11 remaining genes had an increase in gene expression that correlated with a decrease in methylation (Table 2).

**Table 2:** Differentially methylated cytosines (DMCs; *P* < 0.05) that had a negative relationship with gene expression (FDR *P* < 0.05)

		DMC	Gene expression			
Chr. position	Gene	Methyl difference (%)	Log fold change	Fold change	Gene region	CpG island region
chr11.64670967	ATG2A	−6.8	0.62	1.5	Promoter	InterCpG
chrX.107334934	ATG4A	−11.1	0.59	1.5	Promoter	Cpg island
chrX.107334999	ATG4A	−5.3	0.59	1.5	Promoter	Cpg island
chr21.45749947	C21orf2	−6.3	0.43	1.3	3'UTR	North shelf
chrX.30671522	GK	−31.4	0.42	1.3	5'UTR	Cpg island
chrX.30671506	GK	−14.1	0.42	1.3	5'UTR	Cpg island
chr19.5153271	KDM4B	−20.7	0.39	1.3	3'UTR	South shore
chr9.34381797	KIAA1161	−13.1	0.60	1.5	Promoter	South shore
chr4.6641531	MRFAP1	−11.2	0.70	1.6	Promoter	North shore
chr1.145609911	POLR3C	−19.7	0.47	1.4	Promoter	North shore
chrX.20286470	RPS6KA3	−15.3	0.46	1.4	Promoter	Cpg island
chr9.135231749	SETX	−16.2	0.55	1.5	Promoter	South shore
chr8.22409297	SORBS3	3.9	−0.91	−1.9	5'UTR	Cpg island
chr8.22422648^a^	SORBS3	5.0	−0.91	−1.9	Promoter	Cpg island
chr8.22423300	SORBS3	10.6	−0.91	−1.9	5'UTR	Cpg island
chr8.22423343	SORBS3	16.4	−0.91	−1.9	5'UTR	Cpg island
chr8.22422936	SORBS3	16.6	−0.91	−1.9	Promoter	Cpg island
chr8.22422959	SORBS3	17.1	−0.91	−1.9	Promoter	Cpg island
chr8.22422927	SORBS3	17.7	−0.91	−1.9	Promoter	Cpg island
chr8.22423332	SORBS3	20.3	−0.91	−1.9	5'UTR	Cpg island
chr8.22423092	SORBS3	24.4	−0.91	−1.9	Promoter	Cpg island
chr6.56972737	ZNF451	−4.7	0.55	1.5	3'UTR	InterCpG

^a^This position indicates the site validated by site specific methylation sequencing

### Differentially methylated region (DMR) analysis in the promoter, 5′UTR, and 3′UTR regions

To further interrogate changes in methylation, a regional analysis was performed and identified 700 DMRs. Of these, 170 were located in our defined proximal regions (Additional file 3: Table S3). The 170 DMRs were compared with the 99 probes identified from the microarray analysis. We identified one DMR (Chr.8:22,423,530–22,423,569) in the 5′UTR of SORBS3 that demonstrated a negative relationship with gene expression. The DMR was increased by 11.2 % in the obese group.

### SORBS3 validation

**Fig. 3:**
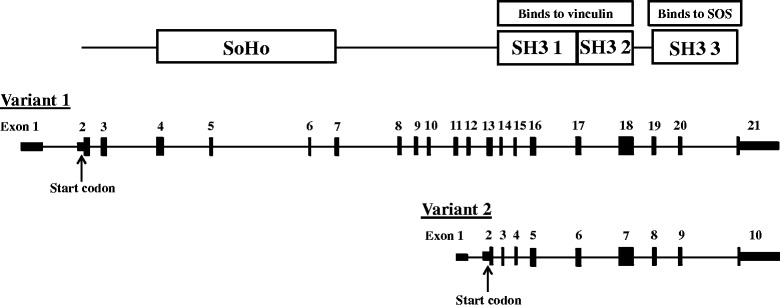
Sorbin and SH3 domain containing 3 (SORBS3) consists of two transcript variants that code for two protein isoforms, vinexin alpha and beta, respectively. Variant 2 (vinexin beta) exons 3–10 are consistent with variant 1 exons 14–21, containing all three SH3 domains. Variant 2 differs by lacking the coding regions for the N-terminal end SoHo domain

**Fig. 4:**
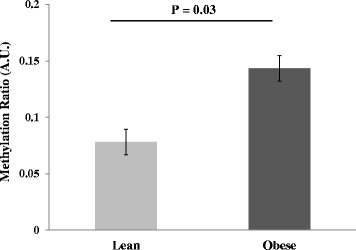
DNA methylation in the promoter of SORBS3 was validated with the site specific sequencing approach

**Fig. 5:**
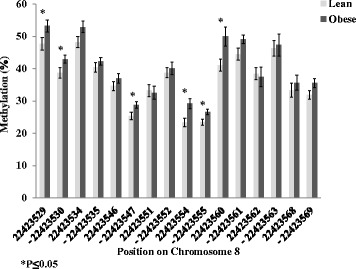
DNA methylation detected using pyrosequencing in the differentially methylated region (DMR) of sorbin and SH3 domain containing 3 (SORBS3) on both the forward and reverse (−) strands

SORBS3 has two transcript variants (variant 1: NM_005775 and variant 2: NM_001018003) as shown in Fig. 3. We used a site-specific sequencing approach to validate a promoter site of variant 2 (Chr.8:22,422,648). The RRBS data had shown a 5 % increase in methylation in the obese compared to the lean participants (Additional file 1: Table S1). Validation using site specific sequencing demonstrated an increase in methylation in the participants with obesity (lean 0.078 ± 0.01 versus obese 0.14 ± 0.03 methylation ratio; *P* = 0.03; Fig. 4). Pyrosequencing of the SORBS3 DMR (Chr.8:22,423,530–22,423,569) in the 5′UTR of variant 2 resulted in an overall increase in methylation, as shown in Fig. 5. Three sites on the forward strand and three on the reverse strand were significantly different (*P* < 0.05) with obesity using the pyrosequencing analysis, which further validated the RRBS findings (Fig. 5). The qRT-PCR confirmed the microarray results (Table 2) demonstrating a decrease in gene expression of SORBS3 in the participants with obesity (fold change −1.4; *P* = 0.01).

### Predicted transcription factor binding analysis

**Fig. 6:**
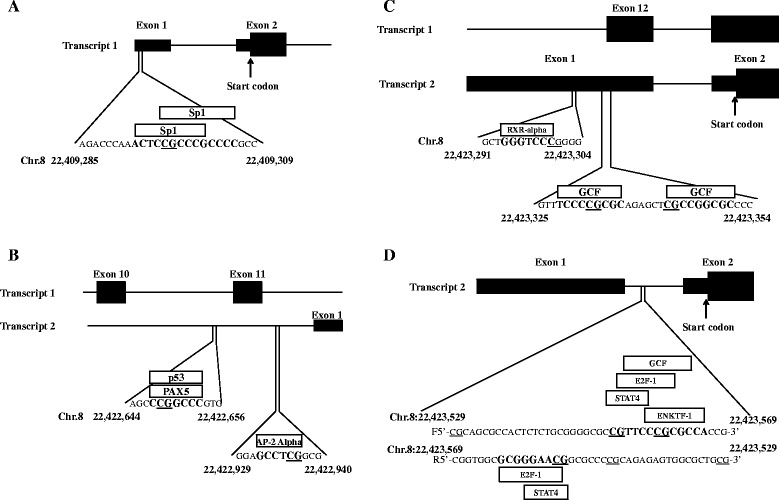
Transcription factor binding analysis. **a** Differentially methylated cytosine (DMC) at chromosome 8 position 22,409,297 is in the 5' untranslated region of sorbin and SH3 domain containing 3 (SORBS3) variant 1. This DMC is within two binding motifs for the transcription factor specificity protein 1 (Sp1). **b** DMCs at chromosome 8 positions 22,422,648 and 22,422,936 are in the promoter region of SORBS3 variant 2. The DMC position 22,422,648 is within a two binding motifs for the transcription factors paired box 5 (PAX5) and tumor protein p53 (p53). The DMC position 22,422,936 is within a binding motif for the transcription factor activating enhancer-binding protein 2-alpha (AP-2 Alpha). **c** DMCs at chromosome 8 positions 22,423,300, 22,423,332, and 22,423,343 are in the 5' untranslated region of SORBS3 variant 2. The DMC position 22,423,300 is within the binding motif for the transcription factor retinoid X receptor, alpha (RXR-alpha). The DMC positions 22,423,332 and 22,423,343 are both within binding motifs for the transcription factor GC binding factor (GCF). **d** The SORBS3 differentially methylated region (DMR) is located at chromosome 8 position 22,423,529–22,423,569 is in the 5' untranslated region of variant 2. On the forward strand, position 22,423,554 is within a binding motif for signal transducer and activator of transcription 4 (STAT4) and positon 22,423,560 is within the binding motif of enkephalin transcription factor 1 (ENKTF-1), E2F transcription factor 1 (E2F-1), and GC binding factor (GCF). On the reverse strand, position 22,423,555 is within the binding motif of E2F-1 and STAT4

To further understand the regulatory role of SORBS3 methylation on transcription, we analyzed the sequences containing DMCs and the DMR using the program PROMO [29]. Transcription factor binding motifs were identified for the following DMC positions: Chr.8:22,409,297- Sp1 (Fig. 6a); Chr.8:22,422,648-p53, Chr.8:22,422,648-PAX5, and Chr.8:22,422,936-AP-2alpha (Fig. 6b); Chr.8:22,423,300-RXR-alpha, Chr.8:22,423,332-GCF, and 22423343-GCF (Fig. 6c). The transcription factor binding motifs identified within the DMR for SORBS3 on Chr.8:22,423,530–22,423,569 were ENKTF-1, STAT4, E2F-1, and GCF (Fig. 6d).

### Correlation analysis

To identify whether the methylation and transcriptomic findings for SORBS3 were driven by body mass index (BMI) or age, Pearson correlation analysis was performed. Of the nine DMCs, five were significantly correlated with BMI and one was significantly correlated with age (Table 3). When comparing the normalized gene expression data with BMI there was a significant correlation (*R*^2^ = 0.288; *P* = 0.022), whereas with age, there was no correlation (*R*^2^ = 0.034; *P* = 0.464).

**Table 3:** Correlation analysis of differentially methylated cytosines (DMCs) sorbin and SH3 domain containing 3 (SORBS3) with body mass index (BMI) and age

		BMI		Age	
Chr.	Position	*R* ^2^	*P* Value	*R* ^2^	*P* Value
8	22,409,297	0.092	NS	0.009	NS
8	22,422,648	0.329	0.013	0.209	NS
8	22,422,927	0.243	0.038	0.169	NS
8	22,422,936	0.264	0.029	0.238	0.040
8	22,422,959	0.169	NS	0.078	NS
8	22,423,092	0.254	0.033	0.017	NS
8	22,423,300	0.199	NS	0.202	NS
8	22,423,332	0.135	NS	0.144	NS
8	22,423,343	0.261	0.036	0.043	NS

*R* ^2^ and *P* values were generated using Pearson correlation

## Discussion

The present study was undertaken to decipher the epigenetic basis of obesity and its associated insulin resistance. DNA methylation in the promoter and untranslated regions (5′ and 3′UTR) have been noted to have regulatory effects on transcription [14–16]. This regulation can be mediated by a single CpG or by a group of CpGs in close proximity to each other [31]. Therefore, in our study, we performed a comprehensive analysis of the sequencing data using both a DMC and DMR approach. To identify obesity-related alterations in gene expression that may be associated with DNA methylation, our study also utilized a transcriptomic approach. Merging across our omic datasets identified sorbin and SH3 domain containing 3 (SORBS3) as a novel obesity gene. SORBS3 is decreased in expression in obesity, and this in part may be due to increased methylation. Moreover, we detected a number of transcription factors whose binding to the differentially methylated sites or regions may contribute to these findings [32].

SORBS3 has two transcript variants that code for the adapter protein vinexin α and β, respectively. Both isoforms have a common C-terminal sequence containing three SRC homology 3 (SH3) domains but differ at the *N*-terminal where vinexin α contains a sorbin homology (SoHo) domain. Vinexin α and β play roles in cell signaling and the cytoskeletal structure [33]. The first two SH3 domains (SH3 1 and SH3 2) are important binding partners for vinculin, which is an actin-binding cytoskeletal protein localized at cell-extracellular matrix (ECM) and cell-cell adhesion sites [34]. It has been shown elsewhere that the upregulation of vinexin α promotes actin stress fiber formation and vinexin β enhanced cell spreading [34]. Our obesity associated decrease in gene expression may suggest a reduced plasticity of cytoskeleton organization. The third SH3 domain (SH3 3) is an important binding partner for the son of sevenless (SOS), a guanine nucleotide exchange factor for Ras and Rac [33]. Vinexin’s interaction with SOS has been implicated to regulate growth-factor-induced signal transduction [33]. For example, a knockdown model of vinexin has been shown to play a key role in the cell’s migratory response during wound healing [35]. The reduction in SORBS3 gene expression seen in our group with obesity may lead to a delayed response in growth-factor signaling.

Additional studies have evaluated vinexin under diseased states. A study using immunohistochemical analyses of vinexin in Otsuka Long Evans Tokushima Fatty (OLETF) rats with hyperinsulinemia and hyperglycemia demonstrated a disorganized pancreatic islet structure [36]. Although abundance of vinexin was not discussed in that study, these findings infer that an obese environment can disrupt typical localization of vinexin within a cell. We have previously shown alterations in cytoskeletal proteins in insulin resistant states [7]. Therefore, we hypothesize that a change in expression of SORBS3 in obesity could be contributing to altered skeletal muscle structure. However, further investigation would be required. Chen et al. found that left ventricles of failing human hearts had a decrease in mRNA for vinexin β, and the disruption of vinexin expression in C57BL/6 mice exaggerated pathological cardiac remodeling and fibrosis [37]. Obesity can lead to cardiovascular changes such as left-ventricular hypertrophy [38, 39]. Although our study found reduced expression of SORBS3 in the *vastus lateralis* of individuals with obesity, it is tempting to speculate that there may be a similar remodeling and fibrotic affect due to vinexin β.

The findings from our previous studies had led to a proposed model of a relationship between inflammation and insulin resistance in skeletal muscle [8]. In this model, chronic inflammation from obesity may induce changes to the extracellular matrix that are reminiscent of fibrosis and alter mechanosignal transduction mediated by cytoskeletal elements [8]. The changes in obesity with SORBS3 expression coding for vinexin may be connected to our proposed model by regulating the plasticity of cytoskeletal elements. Interestingly, if vinexin is a key component to this model, we have identified possible regulation at the level of DNA by differentially methylated sites and regions. Moreover, the mechanism for this regulation could be due to the interaction of these methylation sites with the transcription factors identified in our analyses.

## Conclusions

To our knowledge, this was the first study to examine obesity-related differential DNA methylation in skeletal muscle using RRBS. The design of our epigenomic study not only allowed us to test our specific hypotheses but also generated a novel methylation and transcriptional finding for further investigation. Furthermore our RRBS data can serve as a reference methylome for human skeletal muscle tissue. Despite these strengths, we acknowledged potential limitations that should be considered. There is a difference in age between our groupings that could be a confounding factor in the results presented. We did attempt to reduce this concern by running correlation analysis of age with SORBS3 gene expression and each associated methylation site. Future age-matched studies could elucidate any findings that may have been influenced by this variable. In addition, the potential for false discoveries may be at higher risk since our methylation data remained uncorrected. However, our chances of detecting true biological effects may be increased by the use both DMC and DMR analyses.

Overall, our study identified possible epigenetic influence on differential gene expression in SORBS3 under obese conditions. We identified potential transcriptional regulators; however, follow-up studies of their protein interactions with DNA methylation are necessary to refine the mechanism. Furthermore, the previously mentioned functional studies of vinexin under diseased states have been conducted in rodent models and should be further assessed in humans.

## Abbreviations

ATG2A, autophagy-related 2A; ATG4A, autophagy-related 4A; C21orf2, chromosome 21 open reading frame 2; DMC, differentially methylated cytosine; DMR, differentially methylated region; ECM, extracellular matrix; GK, glycerol kinase; KDM4B, lysine demethylase 4B; MRFAP1, Morf4 family associated protein 1; POLR3C, polymerase (RNA) III (DNA directed) polypeptide C; RPS6KA3, ribosomal protein S6 kinase, 90-kDa, polypeptide 3; RRBS, reduced representation bisulfite sequencing; SETX, senataxin; SORBS3, sorbin and SH3 domain containing 3; UTR, untranslated region; ZNF451, zinc finger protein 451

## Additional files

Additional file 1: Table S1. The 13,130 differentially methylated cytosines (DMCs; *P* < 0.05) that were located within a promoter or untranslated regions (3' and 5'). (XLSX 2880 kb) Additional file 2: Table S2. The probes (*n* = 99) remaining from the microarray data that met the false discovery rate (FDR) correction criteria of *P* < 0.05. (XLSX 68 kb) Additional file 3: Table S3. The 170 differentially methylated regions (DMRs; *P* < 0.05) that were located within a promoter or untranslated regions (3' and 5'). (XLSX 35580 kb)

## References

[1] Ogden CL, Carroll MD, Kit BK, Flegal KM (2014). Prevalence of childhood and adult obesity in the United States, 2011–2012. JAMA.

[2] Guh DP, Zhang W, Bansback N, Amarsi Z, Birmingham CL, Anis AH (2009). The incidence of co-morbidities related to obesity and overweight: a systematic review and meta-analysis. BMC Public Health.

[3] Kahn SE, Hull RL, Utzschneider KM (2006). Mechanisms linking obesity to insulin resistance and type 2 diabetes. Nature.

[4] Abdul-Ghani MA, DeFronzo RA (2010). Pathogenesis of insulin resistance in skeletal muscle. J Biomed Biotechnol.

[5] Patti ME, Butte AJ, Crunkhorn S, Cusi K, Berria R, Kashyap S, Miyazaki Y, Kohane I, Costello M, Saccone R (2003). Coordinated reduction of genes of oxidative metabolism in humans with insulin resistance and diabetes: potential role of PGC1 and NRF1. Proc Natl Acad Sci U S A.

[6] Richardson DK, Kashyap S, Bajaj M, Cusi K, Mandarino SJ, Finlayson J, DeFronzo RA, Jenkinson CP, Mandarino LJ (2005). Lipid infusion decreases the expression of nuclear encoded mitochondrial genes and increases the expression of extracellular matrix genes in human skeletal muscle. J Biol Chem.

[7] Hwang H, Bowen BP, Lefort N, Flynn CR, De Filippis EA, Roberts C, Smoke CC, Meyer C, Hojlund K, Yi Z, Mandarino LJ (2010). Proteomics analysis of human skeletal muscle reveals novel abnormalities in obesity and type 2 diabetes. Diabetes.

[8] Coletta DK, Mandarino LJ (2011). Mitochondrial dysfunction and insulin resistance from the outside in: extracellular matrix, the cytoskeleton, and mitochondria. Am J Physiol Endocrinol Metab.

[9] Fernandez JR, Pearson KE, Kell KP, Bohan Brown MM (2013). Genetic admixture and obesity: recent perspectives and future applications. Hum Hered.

[10] Wang T, Jia W, Hu C. Advancement in genetic variants conferring obesity susceptibility from genome-wide association studies. Front Med. 2014. 10.1007/s11684-014-0373-8 25556696

[11] Egger G, Liang G, Aparicio A, Jones PA (2004). Epigenetics in human disease and prospects for epigenetic therapy. Nature.

[12] Huidobro C, Fernandez AF, Fraga MF (2013). The role of genetics in the establishment and maintenance of the epigenome. Cell Mol Life Sci.

[13] Jeltsch A, Jurkowska RZ (2014). New concepts in DNA methylation. Trends Biochem Sci.

[14] Yu B, Russanova VR, Gravina S, Hartley S, Mullikin JC, Ignezweski A, Graham J, Segars JH, DeCherney AH, Howard BH (2015). DNA methylome and transcriptome sequencing in human ovarian granulosa cells links age-related changes in gene expression to gene body methylation and 3′-end GC density. Oncotarget.

[15] Ling C, Groop L (2009). Epigenetics: a molecular link between environmental factors and type 2 diabetes. Diabetes.

[16] Maussion G, Yang J, Suderman M, Diallo A, Nagy C, Arnovitz M, Mechawar N, Turecki G (2014). Functional DNA methylation in a transcript specific 3′UTR region of TrkB associates with suicide. Epigenetics.

[17] Barres R, Kirchner H, Rasmussen M, Yan J, Kantor FR, Krook A, Naslund E, Zierath JR (2013). Weight loss after gastric bypass surgery in human obesity remodels promoter methylation. Cell Rep.

[18] Alibegovic AC, Sonne MP, Hojbjerre L, Bork-Jensen J, Jacobsen S, Nilsson E, Faerch K, Hiscock N, Mortensen B, Friedrichsen M (2010). Insulin resistance induced by physical inactivity is associated with multiple transcriptional changes in skeletal muscle in young men. Am J Physiol Endocrinol Metab.

[19] DeFronzo RA, Tobin JD, Andres R (1979). Glucose clamp technique: a method for quantifying insulin secretion and resistance. Am J Physiol.

[20] Cusi K, Maezono K, Osman A, Pendergrass M, Patti ME, Pratipanawatr T, DeFronzo RA, Kahn CR, Mandarino LJ (2000). Insulin resistance differentially affects the PI 3-kinase- and MAP kinase-mediated signaling in human muscle. J Clin Invest.

[21] Debodo RC, Steele R, Altszuler N, Dunn A, Bishop JS (1963). On the hormonal regulation of carbohydrate metabolism; studies with C14 glucose. Recent Prog Horm Res.

[22] Gu H, Smith ZD, Bock C, Boyle P, Gnirke A, Meissner A (2011). Preparation of reduced representation bisulfite sequencing libraries for genome-scale DNA methylation profiling. Nat Protoc.

[23] Sun Z, Baheti S, Middha S, Kanwar R, Zhang Y, Li X, Beutler AS, Klee E, Asmann YW, Thompson EA, Kocher JP (2012). SAAP-RRBS: streamlined analysis and annotation pipeline for reduced representation bisulfite sequencing. Bioinformatics.

[24] Xi Y, Li W (2009). BSMAP: whole genome bisulfite sequence MAPping program. BMC Bioinformatics.

[25] Li H, Handsaker B, Wysoker A, Fennell T, Ruan J, Homer N, Marth G, Abecasis G, Durbin R, Genome Project Data Processing S (2009). The sequence alignment/map format and SAMtools. Bioinformatics.

[26] Park Y, Figueroa ME, Rozek LS, Sartor MA (2014). MethylSig: a whole genome DNA methylation analysis pipeline. Bioinformatics.

[27] Wu H, Xu T, Feng H, Chen L, Li B, Yao B, Qin Z, Jin P, Conneely KN (2015). Detection of differentially methylated regions from whole-genome bisulfite sequencing data without replicates. Nucleic Acids Res.

[28] Livak KJ, Schmittgen TD (2001). Analysis of relative gene expression data using real-time quantitative PCR and the 2(−delta delta C(T)) method. Methods.

[29] Messeguer X, Escudero R, Farre D, Nunez O, Martinez J, Alba MM (2002). PROMO: detection of known transcription regulatory elements using species-tailored searches. Bioinformatics.

[30] Hall E, Volkov P, Dayeh T, Bacos K, Ronn T, Nitert MD, Ling C (2014). Effects of palmitate on genome-wide mRNA expression and DNA methylation patterns in human pancreatic islets. BMC Med.

[31] Li S, Garrett-Bakelman FE, Akalin A, Zumbo P, Levine R, To BL, Lewis ID, Brown AL, D’Andrea RJ, Melnick A, Mason CE (2013). An optimized algorithm for detecting and annotating regional differential methylation. BMC Bioinformatics.

[32] Attwood JT, Yung RL, Richardson BC (2002). DNA methylation and the regulation of gene transcription. Cell Mol Life Sci.

[33] Kioka N, Ueda K, Amachi T (2002). Vinexin, CAP/ponsin, ArgBP2: a novel adaptor protein family regulating cytoskeletal organization and signal transduction. Cell Struct Funct.

[34] Kioka N, Sakata S, Kawauchi T, Amachi T, Akiyama SK, Okazaki K, Yaen C, Yamada KM, Aota S (1999). Vinexin: a novel vinculin-binding protein with multiple SH3 domains enhances actin cytoskeletal organization. J Cell Biol.

[35] Kioka N, Ito T, Yamashita H, Uekawa N, Umemoto T, Motoyoshi S, Imai H, Takahashi K, Watanabe H, Yamada M, Ueda K (2010). Crucial role of vinexin for keratinocyte migration in vitro and epidermal wound healing in vivo. Exp Cell Res.

[36] Yamauchi M, Sudo K, Ito H, Iwamoto I, Morishita R, Murai T, Kajita K, Ishizuka T, Nagata K (2013). Localization of multidomain adaptor proteins, p140Cap and vinexin, in the pancreatic islet of a spontaneous diabetes mellitus model, Otsuka Long-Evans Tokushima Fatty rats. Med Mol Morphol.

[37] Chen K, Gao L, Liu Y, Zhang Y, Jiang DS, Wei X, Zhu XH, Zhang R, Chen Y, Yang Q (2013). Vinexin-beta protects against cardiac hypertrophy by blocking the Akt-dependent signalling pathway. Basic Res Cardiol.

[38] Vasan RS (2003). Cardiac function and obesity. Heart.

[39] Cuspidi C, Rescaldani M, Sala C, Grassi G (2014). Left-ventricular hypertrophy and obesity: a systematic review and meta-analysis of echocardiographic studies. J Hypertens.

